# Rational Design of 1-D Co_3_O_4_ Nanofibers@Low content Graphene Composite Anode for High Performance Li-Ion Batteries

**DOI:** 10.1038/srep45105

**Published:** 2017-03-27

**Authors:** Su-Ho Cho, Ji-Won Jung, Chanhoon Kim, Il-Doo Kim

**Affiliations:** 1 Department of Materials Science and Engineering, Korea Advanced Institute of Science and Technology (KAIST), 291 Daehak-ro, Yuseong-gu, Daejeon 34141, Republic of Korea

## Abstract

Cobalt oxide that has high energy density, is the next-generation candidate as the anode material for LIBs. However, the practical use of Co_3_O_4_ as anode material has been hindered by limitations, especially, low electrical conductivity and pulverization from large volume change upon cycling. These features lead to hindrance to its electrochemical properties for lithium-ion batteries. To improve electrochemical properties, we synthesized one-dimensional (1-D) Co_3_O_4_ nanofibers (NFs) overed with reduced graphene oxide (rGO) sheets by electrostatic self-assembly (Co_3_O_4_ NFs@rGO). The flexible graphene oxide sheets not only prevent volume changes of active materials upon cycling as a clamping layer but also provide efficient electrical pathways by three-dimensional (3-D) network architecture. When applied as an anode for LIBs, the Co_3_O_4_ NFs@rGO exhibits superior electrochemical performance: (i) high reversible capacity (615 mAh g^−1^ and 92% capacity retention after 400 cycles at 4.0 A g^−1^) and (ii) excellent rate capability. Herein, we highlighted that the enhanced conversion reaction of the Co_3_O_4_ NFs@rGO is attributed to effective combination of 1-D nanostructure and low content of rGO (~3.5 wt%) in hybrid composite.

Lithium-ion batteries (LIBs) have been widely used as the energy source of various devices including portable electronics, electric vehicles (EVs) and energy storage system (ESS) due to their high power, energy density and clean system^1^,^2^,^3^. However, the performance of LIBs is still falling short of the level required for large-scale applications. It pushes the engineers to develop high capacity electrode materials, especially anode, for replacing commercial anode material (e.g. graphite with low specific capacity of 372 mAh g^−1^). Transition metal oxides (TMOs) introduced by Tarascon’s group are next-generation anode materials thanks to their high theoretical capacity based on conversion reaction^4^,^5^,^6^,^7^. Among them, cobalt oxide (Co_3_O_4_) is an emerging candidate because of its high theoretical capacity (890 mAh g^−1^), based on the conversion reaction. Typically, during discharge process, Co_3_O_4_ can react with 8 moles of lithium ions (Li^+^) and 8 moles of electrons and form cobalt (Co) metal nanograins which is distributed in lithium oxide (Li_2_O) matrix. The conversion reaction of Co_3_O_4_ is represented in the following equation.


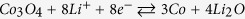


With many Li^+^ transport and electron transfer, higher theoretical capacity can be obtained from Co_3_O_4_ compared to other carbonaceous materials.

Nevertheless, practical use of Co_3_O_4_ has been frustrated by the following main challenges: large volume changes of Co_3_O_4_ (~300%) upon cycling, low electrical conductivity of Co_3_O_4_, and formation of insulative Li_2_O matrix due to the conversion reaction. In order to address these issues, researchers have often employed nano-engineering to develop Co_3_O_4_/carbon nanocomposites such as Co_3_O_4_ nanoparticles (NPs)/graphene^8^, Co_3_O_4_ NPs/carbon tube^9^ and Co_3_O_4_ nanowires (NWs)/graphene frame^10^. Much research has shown that the graphene greatly provides facile passage for electrons and flexible clamping component for Co_3_O_4_^11^,^12^,^13^. However, Co_3_O_4_/graphene composites developed so far require large amount of graphene for stable cycling in LIBs, which sacrifices the actual capacity of Co_3_O_4_. Thus, we suggested desirable design for hybrid nanocomposite architecture of Co_3_O_4_ and graphene.

One-dimensional (1-D) NFs, in common with other nanostructures, have large electrode/electrolyte interface area with volume-strain relaxation which can prevent the volume expansion during charge/discharge process. Moreover, these NFs have larger electrochemical active area because they are less likely to undergo aggregation than nanoparticles^14^,^15^,^16^,^17^. In general, large amount of graphene is required to cover nanoparticles with large specific surface area. Meanwhile, it is possible to coat entire surfaces of NFs with very low-content of graphene.

In this work, the hybrid Co_3_O_4_ NFs@rGO offer significant benefits that are enumerated briefly as follows: 1-D reinforcement scaffold of the Co_3_O_4_ NFs for fast Li^+^ diffusion, large area for electrode/electrolyte interface, continuous electric contact and volume accommodation, and flexible yet robust graphene sheets directly coated on the Co_3_O_4_ NFs for highly electrically conductive networks before and after conversion reactions. Small amount of the rGO greatly improved the performance of nanocomposite, synergistic effect of which on conversion reaction is emphasized.

## Results

### Synthesis of 1-D Co_3_O_4_ NFs and Co_3_O_4_ NFs@rGO

**Figure 1:**
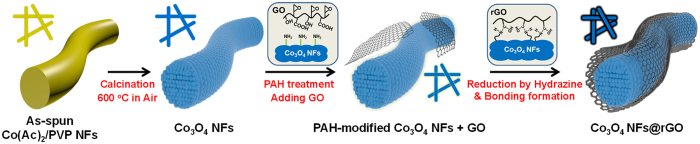
The schematic illustration of synthesis for the Co_3_O_4_ NFs and the Co_3_O_4_ NFs@rGO.

As shown in Fig. 1, the 1-D Co_3_O_4_ NFs were fabricated via electrospinning method. As-spun Co precursor/polymer composite NFs (as-spun Co(Ac)_2_/PVP NFs) were achieved after the electrospinning. In subsequent calcination step at 600 °C in air, residual solvent and polymer were burned out and Co precursor was oxidized to Co_3_O_4_, resulting in the formation of 1-D Co_3_O_4_ NFs. Finally, to cover the surface of the Co_3_O_4_ NFs with highly conducting graphene sheets, we employed chemical solution method based on electrostatic interaction and chemical bonds^18^. The as-prepared Co_3_O_4_ NFs were placed in poly(allylamine hydrochloride) solution (PAH solution), where hydroxyl group (-OH) on Co_3_O_4_ NFs was modified to amine group (-NH_2_). These amine groups on the PAH-modified Co_3_O_4_ NFs have the electrostatic attraction with the epoxy groups of graphene oxide (GO), and ring opening reaction between the PAH-modified Co_3_O_4_ NFs and GO occurs. After reduction of the GO with hydrazine, strong chemical bonds between the Co_3_O_4_ NFs and reduced GO (rGO) were generated, leading to the rGO sheets wrapped Co_3_O_4_ NFs (Co_3_O_4_ NFs@rGO).

### Characteristics of Co_3_O_4_ NFs and Co_3_O_4_@rGO NFs

**Figure 2:**
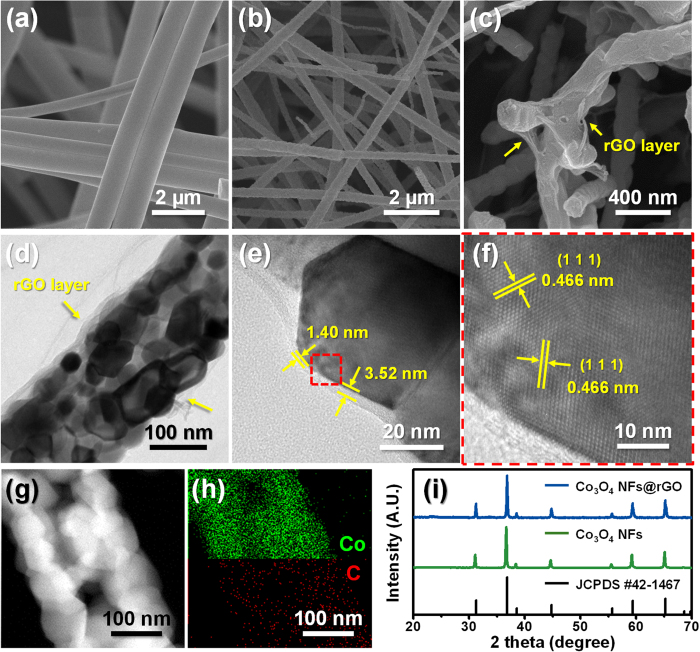
SEM images of (**a**) the as-spun NFs, (**b**) the Co_3_O_4_ NFs, and (**c**) the Co_3_O_4_ NFs@rGO. (**d–f**) HRTEM images of the Co_3_O_4_ NFs@rGO. (**g**) STEM image of the Co_3_O_4_ NFs@rGO. (**h**) EDS-mapping image for atomic distribution of Co and C analyzed from the STEM image in (**g**). (**i**) XRD patterns of the Co_3_O_4_ NFs@rGO, the Co_3_O_4_ NFs and JCPDS #42-1467.

Morphological features of the Co_3_O_4_ NFs@rGO are represented in Fig. 2. The as-spun Co(Ac)_2_/PVP NFs with smooth and uniform surface were observed in Fig. 2a. During subsequent calcination changing the as-spun NFs into the Co_3_O_4_ NFs, the oxidation with shrinkage by burning of residual solvent and polymer was taken place. The calcined Co_3_O_4_ NFs were formed in a diameter of 150 to 200 nm (Fig. 2b). After graphene wrapping process, it is clearly observed that the rGO layers are homogeneously coated on the surface of the Co_3_O_4_ NFs (Fig. 2c). The internal morphology and phase of the Co_3_O_4_ NFs and the Co_3_O_4_ NFs@rGO were confirmed by transmission electron microscopy (TEM) (Fig. 2d–f and Fig. S1, see Supplementary Information). The high-resolution TEM (HRTEM) images of the Co_3_O_4_ NFs@rGO is shown in Fig. 2e,f, showing that polycrystalline Co_3_O_4_ grains connected together were uniformly covered by the ultrathin rGO sheets (~3 nm) (Fig. 2d,e). The lattice fringe of the Co_3_O_4_ in the Co_3_O_4_ NFs@rGO is 4.66 Å, which is well matched with the spacing of Co_3_O_4_ (111) planes (JCPDS PDF#43-1467) (Fig. 2f). The scanning TEM-energy dispersive spectroscopic (STEM-EDS) mapping analysis for the Co_3_O_4_ NFs@rGO confirms homogeneous atomic distribution of Co and C in the 1-D scaffold (Fig. 2g,h).

To check exact content of carbon in the Co_3_O_4_ NFs@rGO, we conducted element analysis (EA). From the EA results, the concentration of carbon was measured to be only 3.56 wt% (Table S1). This value indicates very small amount of carbon in the Co_3_O_4_ NFs@rGO. Even using minimum amount of carbon to anode, rGO directly coated on Co_3_O_4_ NFs enhances the electrical conductivity and provides high capacity for the Co_3_O_4_ NFs. X-ray diffraction (XRD) patterns show that the Co_3_O_4_ NFs and the Co_3_O_4_ NFs@rGO have the single phase spinel crystal structure for Co_3_O_4_ phase (Fig. 2i). The XRD patterns exhibit additional peaks at 2θ = 31.3°, 36.8°, 38.5°, 44.8°, 55.7°, 59.4° and 65.2°, which are attributed to scattering from the (220), (311), (222), (400), (422), (511) and (440) lattice planes of cubic spinel Fd-3m Co_3_O_4_ phase (JCPDS PDF#43-1467), respectively. Moreover, any peak change was not observed in the Co_3_O_4_ NFs@rGO, indicating that the crystal structure of the Co_3_O_4_ NFs is not affected during chemical-solution based graphene wrapping process.

**Figure 3:**
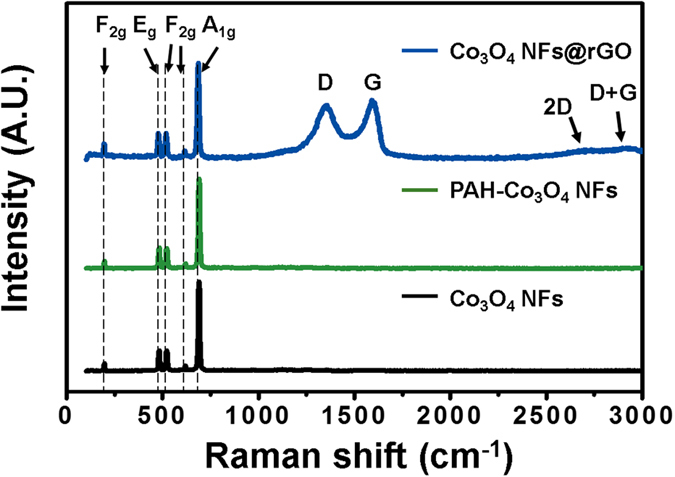
Raman spectra of Co_3_O_4_ NFs@rGO, PAH-treated Co_3_O_4_ NFs and Co_3_O_4_ NFs.

In order to investigate the characteristics of two-dimensional (2-D) graphene layer, Raman spectroscopy analysis was performed. In Raman spectra, five characteristic bands at 195, 478, 517, 618, and 687 cm^−1^ are discovered in common with three samples (the Co_3_O_4_ NFs, PAH-treated Co_3_O_4_ NFs and the Co_3_O_4_ NFs@rGO) (Fig. 3). The band at 195 cm^−1^ (F_2g_) exhibits CoO_6_ scissoring vibration and other bands at 478, 517, 618 and 687 cm^−1^ are assigned to E_g_, 2F_2g_ and A_1g_, that exhibit Co-O symmetric stretching vibration, respectively^19^. In the Co_3_O_4_ NFs@rGO, two specific bands were detected at 1356 and 1590 cm^−1^, which correspond to the D and G band, respectively, confirming that the rGO sheets were formed in the Co_3_O_4_ NFs@rGO^20^. Also, the Raman analysis clearly shows that any structural changes of the Co_3_O_4_ NFs did not occur during graphene wrapping step through any changes in peaks of Co_3_O_4_.

### Electrochemical reaction with Li^+^ of the Co_3_O_4_ NFs and Co_3_O_4_ NFs@rGO

**Figure 4:**
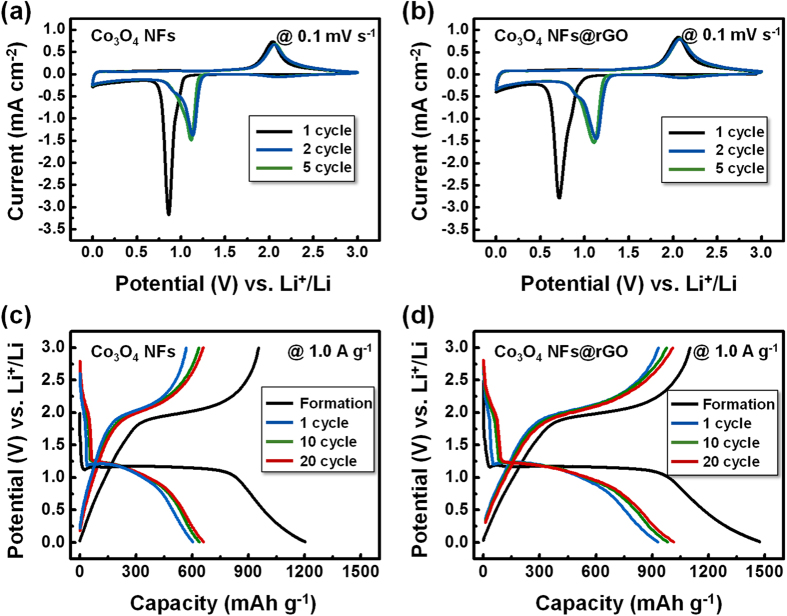
Cyclic voltammetric curves of (**a**) the Co_3_O_4_ NFs and (**b**) the Co_3_O_4_ NFs@rGO tested at a scan rate 0.1 mV s^−1^. Galvanostatic charge-discharge curves of (**c**) the Co_3_O_4_ NFs and (**d**) the Co_3_O_4_ NFs@rGO at a current density of 1.0 A g^−1^.

The electrochemical performances of the Co_3_O_4_ NFs and the Co_3_O_4_ NFs@rGO were investigated to check their potential as high-performance anode materials for LIBs. For the electrochemical data, all capacities in this work were based on the weight of all active materials including rGO. The cyclic voltammetric (CV) curves and galvanostatic charge-discharge curves of the Co_3_O_4_ NFs and the Co_3_O_4_ NFs@rGO are shown in Fig. 4. In Fig. 4a,b the CV graphs show large cathodic peaks at 0.86 V for the Co_3_O_4_ NFs and 0.72 V for the Co_3_O_4_ NFs@rGO in first cycle. These peaks are attributed to the formation of solid-electrolyte interphase (SEI) layer on the surface of active materials. After the 1st cycle, large cathodic peak at 1.2 V is ascribed to the reduction of Co_3_O_4_ by the conversion reaction. The anodic peaks are observed at 2.0 V for both of electrodes, which is ascribed to the oxidation from Co metal nanograins to Co_3_O_4_^21^,^22^. These reaction peaks were well-matched with potentials represented with the plateaus in charge-discharge curves (Fig. 4c,d). The charge-discharge behaviors in the formation cycle, 1st, 10th and 20th cycle for the Co_3_O_4_ NFs and the Co_3_O_4_ NFs@rGO were observed with voltage window between 0.01 and 3.0 V at current densities of 0.1 A g^−1^ for formation cycle and 1.0 A g^−1^ after formation cycle. The discharge capacity in formation cycle of the Co_3_O_4_ NFs@rGO shows higher capacity of 1474 mAh g^−1^, than the Co_3_O_4_ NFs (1202 mAh g^−1^). The irreversible capacity losses of both the Co_3_O_4_ NFs and the Co_3_O_4_ NFs@rGO were estimated to be 25% of the initial discharge capacity.

## Discussion

**Figure 5:**
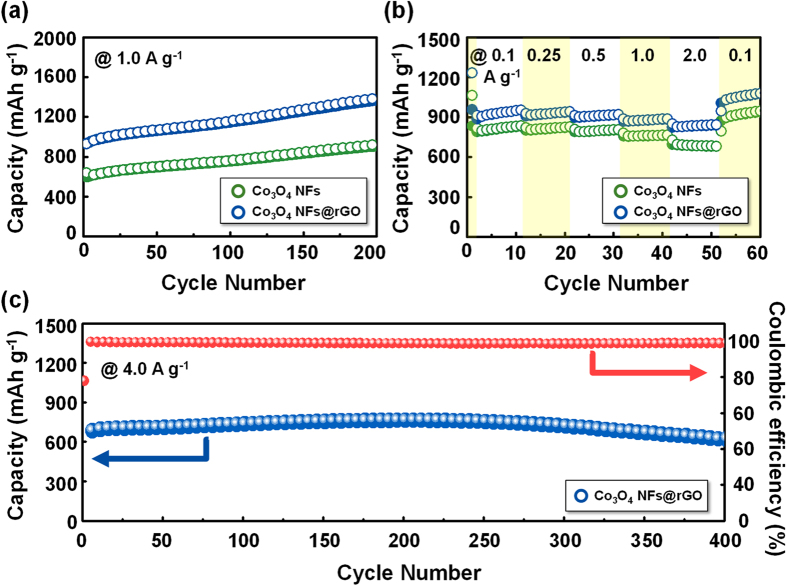
Electrochemical performances of the Co_3_O_4_ NFs and the Co_3_O_4_ NFs@rGO. (**a**) cycle performances of the Co_3_O_4_ NFs and the Co_3_O_4_ NFs@rGO at a current density of 1.0 A g^−1^; (**b**) rate capabilities of the Co_3_O_4_ NFs and the Co_3_O_4_ NFs@rGO at various current densities between 0.1 and 2.0 A g^−1^; (**c**) cycle performance for long-term stability of the Co_3_O_4_ NFs@rGO at high current density of 4.0 A g^−1^.

In general, Co_3_O_4_ converts to the Co metal nanograins dispersed in Li_2_O matrix after conversion reaction with 8 moles of Li^+^ per one mole of Co_3_O_4_. To investigate stability of the the Co_3_O_4_ NFs and the Co_3_O_4_ NFs@rGO toward such severe reaction with Li^+^, cycling performances and rate-capability were evaluated (Fig. 5a–c). In Fig. 5a, the Co_3_O_4_ NFs show the discharge capacity of 639.4 mAh g^−1^ in first cycle at a current density of 1.0 A g^−1^, whereas the discharge capacity of the Co_3_O_4_ NFs@rGO is 933.6 mAh g^−1^ at the same current. For understanding of how the 1-D nanostructures influence their cyclability, we compared the zero-dimensional (0-D) Co_3_O_4_ NPs (control sample) with the 1-D Co_3_O_4_ NFs and the 1-D Co_3_O_4_ NFs@rGO (Fig. S3,SI). Both of the Co_3_O_4_ NFs and the Co_3_O_4_ NFs@rGO show high capacities that could be maintained up to 200th cycle, whereas the Co_3_O_4_ NPs were rapidly degraded within 30 cycles. Furthermore, after 200th cycle, the Co_3_O_4_ NFs and the Co_3_O_4_ NFs@rGO have 924.2 mAh g^−1^ and 1370.8 mAh g^−1^ at a current density of 1.0 A g^−1^, respectively. Figure 5b shows rate capabilities of the Co_3_O_4_ NFs and the Co_3_O_4_ NFs@rGO between 0.1 and 2.0 A g^−1^. At all of current densities, the Co_3_O_4_ NFs@rGO show much higher capacities than pristine Co_3_O_4_ NFs. In addition, the Co_3_O_4_ NFs@rGO exhibit high electrochemical performance that shows first discharge capacity of 669 mAh g^−1^ and 400th discharge capacity of 615 mAh g^−1^ (cycle retention of 92%) at a high current density of 4.0 A g^−1^ (Fig. 5c). It is noticeable that a low amount of rGO (~3.5 wt% in composite) is enough to improve electrochemical performance along with 1-D structural effect in a complementary manner. This amount of carbon is overwhelmingly lower than previously reported values (Table S2).

**Figure 6:**
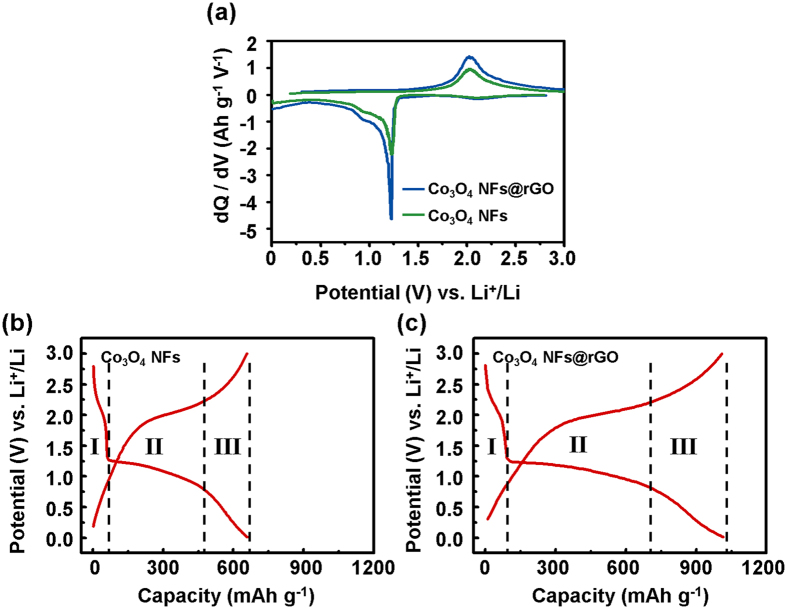
(**a**) The differential capacity (*dQ/dV*) vs. potential (*V*) profiles after 20th cycle at a current density of 1.0 A g^−1^; Galvanostatic charge-discharge curves of (**b**) the Co_3_O_4_ NFs and (**c**) the Co_3_O_4_ NFs@rGO after 20th cycle at a current density of 1.0 A g^−1^.

In Fig. 5, the Co_3_O_4_ NFs and the Co_3_O_4_ NFs@rGO tend to increase the capacity as the cycle progresses. Such increase in capacity of the Co_3_O_4_ NFs may be due to an activated formation of gel-like polymeric film on Co metal surface^23^. Interestingly, as shown in Fig. 5, the capacity of Co_3_O_4_ NFs@rGO has been increased through the graphene wrapping process, and also its capacity is higher than the theoretical capacity of Co_3_O_4_ NFs@rGO 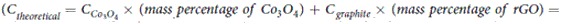

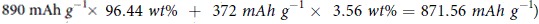
. These extra capacities can be explained by the interfacial Li^+^ storage that represents the formation of gel-like polymeric film and the pseudo-capacitive property on Co metal grains. The gel-like polymeric film is the reversible product from the side reaction of electrolyte decomposition on the surface of Co metal grain at low voltage during discharge process, and this film provides extra capacity for lithium storage^24^,^25^. In addition, the Co metal grain which came from conversion reaction can allow extra Li^+^ adsorption site by metallic pseudo-capacitive property^26^,^27^,^28^. In consideration of such extra charge, unlike the pristine Co_3_O_4_ NFs, the Co_3_O_4_ NFs@rGO provide the fast electron pathway to form negatively charged Co metal grains for effective Li^+^ adsorption on their surface. Figure 6a shows the differential capacity plots for Co_3_O_4_ NFs and Co_3_O_4_ NFs@rGO after 20^th^ cycle at a current density of 1.0 A g^−1^. The broad cathodic peak at around 2.2 V is caused by Li^+^ insertion to Co_3_O_4_NFs, which corresponds to region I in Fig. 6b,c. The peak at 1.23 V is the reduction of Co_3_O_4_ to Co metal nanograins (conversion reaction). These peaks are positioned at same potential for both samples, because the plateau appears at the same voltage in Fig. 6b,c. In Fig. 6b,c there are the regions I and II that are related to Li^+^ insertion and conversion of Co_3_O_4_ with Li^+^ and the region III is relevant to the interfacial Li^+^ storage ability of active materials occurred in solid-liquid interface with Li^+^ adsorption^29^,^30^,^31^. Furthermore, the *dQ/dV* value of Co_3_O_4_ NFs@rGO at 1.23 V is twice that of Co_3_O_4_ NFs. The broad cathodic peak at around 1.0 V is caused by the interfacial lithium ion storage of electrolyte decomposition on Co metal nanograin and pseudo-capacitive property of metal grains. For the peak at around 1.0 V, the *dQ/dV* value of Co_3_O_4_ NFs@rGO is 1.5 times higher compared with the Co_3_O_4_ NFs. Based on these results, the Co_3_O_4_ NFs@rGO proceed the same reaction compared to Co_3_O_4_ NFs, but the rGO layers promote the degree of Li^+^ interfacial storage reaction in all cases of Li^+^ insertion, conversion, and interfacial adsorption.

**Figure 7:**
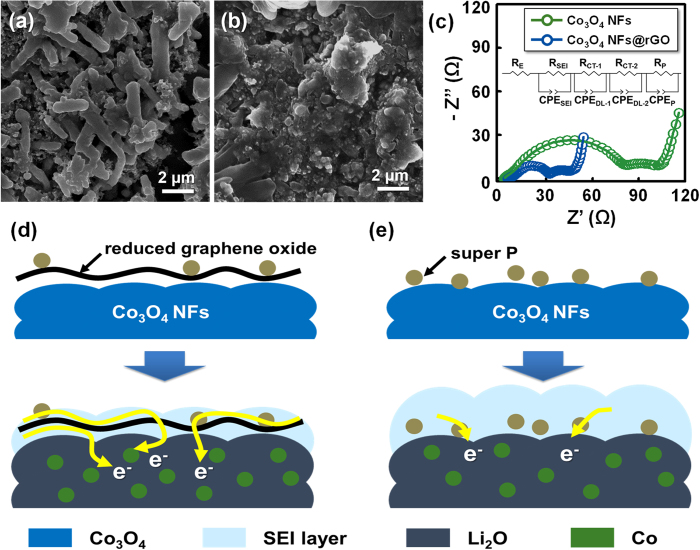
*Ex-situ* scanning electron microscopy images of (**a**) the Co_3_O_4_ NFs@rGO and (**b**) the Co_3_O_4_ NFs after 100th cycle at a current density of 1.0 A g^−1^; (**c**) Nyquist plots of Co_3_O_4_ NFs and Co_3_O_4_ NFs@rGO after the 100th discharge, presented with fitted curves; The illustration of expected mechanism for electron transfer on (**d**) Co_3_O_4_ NFs@rGO and (**e**) Co_3_O_4_ NFs.

To further understand the effect of graphene layer wrapping, *ex-situ* SEM analysis for the Co_3_O_4_ NFs@rGO and the Co_3_O_4_ NFs were carried out after 100th cycle (Fig. 7a,b). The surface state of the delithiated-Co_3_O_4_ NFs@ rGO was observed, indicating that the nanocomposite structure could be intactly preserved with formation of stable SEI layer (Fig. 7a), whereas the SEI layer of the Co_3_O_4_ NFs was conspicuously and irregularly generated (Fig. 7b). To further investigate resistance in the nanocomposites, electrochemical impedance spectroscopy (EIS) measurement was conducted. Figure 7c shows the Nyquist plots of the Co_3_O_4_ NFs and the Co_3_O_4_ NFs@rGO after the 100th cycle and the equivalent model corresponding to the EIS model. The impedance values of each impedance component calculated from the EIS data are represented in Table S3. R_E_, R_SEI_, R_CT-1_, R_CT-2_ and R_P_ are the ohmic resistance of the cell, the SEI resistance, the charge transfer resistances, and the phase transformation resistance, respectively. In the case of the Co_3_O_4_ NFs@rGO, the SEI resistance was reduced by 37.7%, compared to the Co_3_O_4_ NFs. Also, the charge transfer resistance of Co_3_O_4_ NFs@rGO (18.26 Ω) is much smaller than that of Co_3_O_4_ NFs (68.68 Ω). Therefore, it is believed that the low-content rGO layer on Co_3_O_4_ NFs is significantly beneficial to enable smaller polarization for LIBs. Here, we proposed the simple reaction models during discharge process for the Co_3_O_4_ NFs@rGO and the Co_3_O_4_ NFs (Fig. 7d,e). In the case of the Co_3_O_4_ NFs surrounded by SEI and super P (carbon additive), electrons may not efficiently transfer between electrode materials (Fig. 7e). Meanwhile, for the Co_3_O_4_ NFs@rGO, the 2-D rGO layers not only greatly suppress the formation of insulating SEI layer, but also give facile passage for electron. More importantly, after the lithiation, the rGO can be more effective electron bridge between the discharge products (Co NPs in Li_2_O matrix) (Fig. 7d). From the point of view of the Li^+^ interfacial storage, the Co_3_O_4_ NFs@rGO is much suitable compared to Co_3_O_4_ NFs without such conducting route. These envisioned mechanisms can be supported by higher capacity of the Co_3_O_4_ NFs@rGO than that of the Co_3_O_4_ NFs in Fig. 5a,b. Based on the data above and our interpretation, it can be thought that combination of 1-D nanostructure and rGO-wrapping is appropriate for desirably designed electrode architecture, strategy of which will be an explicit direction for conversion-based LIBs.

In summary, we designed the ultra-thin rGO layer wrapped 1-D Co_3_O_4_ NFs as high performance anode for LIBs, which were simply synthesized via an electrospinning and subsequent self-assembly wrapping of graphene sheets on Co_3_O_4_ NFs. 1-D nanostructure of Co_3_O_4_ NFs not only overcome the alleviation of pulverization upon cycling, but also provide the fast Li^+^ diffusion and continuous electric contact. Moreover, the rGO layer, which exists as small percentage of carbon within Co_3_O_4_, provides high electric conductivity to Co_3_O_4_ NFs for high specific capacity, rendering favorable conversion reaction and Li^+^ interfacial storage. The Li anode electrode with Co_3_O_4_ NFs@rGO delivered a relatively high reversible capacity of 615 mAh g^−1^ with stable capacity retention of 92% after 400th cycle at the high current density of 4.0 A g^−1^. Through above results, the reduced graphene oxide sheets wrapped Co_3_O_4_ NFs were suggested as high performance anode for LIBs.

## Methods

### Materials

Cobalt (ii) acetate tetrahydrate (Co(OAc)_2_·4H_2_O, Sigma-Aldrich), polyvinylpyrrolidone (PVP, Mw~1,300,000, Sigma-Aldrich), *N,N*-dimethylformaimde (DMF, 99.8%, Sigma-Aldrich), GO solution (2 ml mg^−1^, Sigma-Aldrich) and poly(allylamine hydrochloride) (PAH, M_w_~900,000, Sigma-Aldrich) were prepared for synthesis of Co_3_O_4_ NFs@rGO. Super P (Alfa Aesar), carboxymethylcellulose sodium salt (CMC, Sigma-Aldrich) and poly(acrylic acid) solution (PAA solution, Sigma-Aldrich, 35 wt.% in H_2_O) were purchased for the electrochemical measurements.

### Synthesis of Co_3_O_4_ NFs

The Co_3_O_4_ NFs were prepared by an electrospinning process and subsequent calcination. 1.5 g of Co(OAc)_2_·4H_2_O and 0.5 g of PVP were dissolved in 4 g of DMF. Electrospinning solution was stirred at 500 rpm for 24 h. The solution was transferred into a plastic syringe equipped with stainless needle. The needle gauge was 25 G. The distance between tip of needle and collector was maintained to 15 cm. The applied voltage on the needle tip was DC 15.0 kV. The solution was pulled out with the flow rate of 25 μl min^−1^. After electrospinning, collected as-spun Co(Ac)_2_/PVP NFs were heated at 600 °C with heating rate of 5 °C min^−1^ for 1 h in air atmosphere for removing polymer and crystallizing Co-precursor to Co_3_O_4_.

### PAH functionalization and graphene wrapping

For graphene wrapping, firstly, 0.25 g PAH was dissolved in 50 ml of distilled water. Then, 0.1 g of the Co_3_O_4_ NFs was added in PAH solution. The surface of the Co_3_O_4_ NFs was modified to amine group by PAH. After magnetic stirring for 2 h, the PAH-treated Co_3_O_4_ NFs were washed three times with distilled water. After that, the PAH-teated Co_3_O_4_ NFs were dried at 50 °C for overnight. For preparing graphene solution, GO was dissolved in distilled water up to 66.3 μg ml^−1^. Sequentially, the PAH-treated Co_3_O_4_ NFs were added into the GO solution, and the mixed solution was stirred at 200 rpm for 12 h. After electrostatic assembly between the PAH-treated Co_3_O_4_ NFs and the GO, to reduce the GO, 0.5 g of hydrazine monohydrate was added into the solution and stirred at 200 rpm for 2 h. Finally, several washing and drying steps were conducted.

### Material characterization

The morphologies of the Co_3_O_4_ NFs and the Co_3_O_4_ NFs@rGO were confirmed *via* scanning electron microscopy (SEM, XL-30 SFEG, Philips). The field emission transmission electron microscope (FE-TEM, Tecnai G2 F30 S-Twin, FEI, Netherlands) was used to check the lattice spacing of Co_3_O_4_ and the thickness of graphene layer. The energy-dispersive spectrometry (EDS) mapping of scanning transmission electron microscope (STEM) shows the distribution of carbon, cobalt and oxygen atoms on NFs. The x-ray diffraction (XRD) pattern of Co_3_O_4_ NFs and Co_3_O_4_ NFs@rGO was carried out using X-Ray diffraction-meter (D/MAX-2500, Rigaku, Japan). The range of measured X-ray diffraction angle was 2θ = 20–70° using Cu-Kα (λ = 1.54 Å) radiation. The amount of carbon component of the Co_3_O_4_ NFs@rGO was analyzed by element analyzer (FLASH 2000 series, Thermo Scientific). To investigate the graphene sheets, Raman spectroscopy (Aramis, Horiba Jobin Yvon, France) was used.

### Electrochemical measurements

To measure electrochemical performance, active materials were mixed with Super P and binders. The binders were used with CMC solution and PAA solution. The ratio of active material (Co_3_O_4_ NFs or Co_3_O_4_ NFs@rGO), super P, CMC solution and PAA solution was 75:15:5:5 in weight. Mixed slurry was coated on Cu-foil with a thickness of 90 μm. Sequentially, the coated Cu-foil was dried at 50 °C for 20 min for removing solvents. For evaporation of moisture and activation of binder, the coated Cu-foil was dried in vacuum oven at 150 °C for 2 h. The obtained anode was assembled to CR2032 coin-type cell in an argon gas filled glove box. The lithium metal was used as counter electrode. The other components of coin cell were Celgard^®^ 2400 as separator, 1.3 M of LiPF_6_ dissolved in EC/DEC = 3/7 (v/v) +10.0 wt% FEC as electrolyte where EC is ethylene carbonate, DEC is diethyl carbonate, and FEC is fluoroethylene carbonate. Loading mass of active material on Cu-foil was 0.88 ± 0.01 mg cm^−2^. Current-Voltage curve was measured at Wonatech WBCS3000 in the voltage window of 0.01–3.0 V and a scan rate of 0.5 mV s^−1^. Galvanostatic charge/discharge process was carried out by Maccor series 4000 in the voltage window of 0.01–3.0 V. Electrochemical impedance spectroscopy (EIS) was performed to investigate the impedance change after 100th cycle with one channel potentiostat (ZIVE SP1 potentiostat, Wonatech, Korea) by applying a sine wave of 10 mV in the frequency range of 10^5^–0.01 Hz.

## Additional Information

**How to cite this article:** Cho, S.-H. *et al*. Rational Design of 1-D Co_3_O_4_ Nanofibers@Low content Graphene Composite Anode for High Performance Li-Ion Batteries. *Sci. Rep.* **7**, 45105; doi: 10.1038/srep45105 (2017).

**Publisher's note:** Springer Nature remains neutral with regard to jurisdictional claims in published maps and institutional affiliations.

## Supplementary Material

Supplementary Information
