# Supplementary material for: Rational Design of 1-D Co3O4 Nanofibers@Low content Graphene Composite Anode for High Performance Li-Ion Batteries
Source: Sci Rep. 2017 Mar 27;7:45105. doi: 10.1038/srep45105 (PMC5366863; doi:10.1038/srep45105)
Supplement: Supplementary Information [file srep45105-s1.doc]

Supplementary Information

**Rational Design of 1-D Co_3_O_4_ Nanofibers@Low content Graphene Composite Anode for High Performance Li-Ion Batteries**

By Su-Ho Cho^1^, Ji-Won Jung^1^, Chanhoon Kim^1^, and Il-Doo Kim^1*^

**[Figure S1]**

**
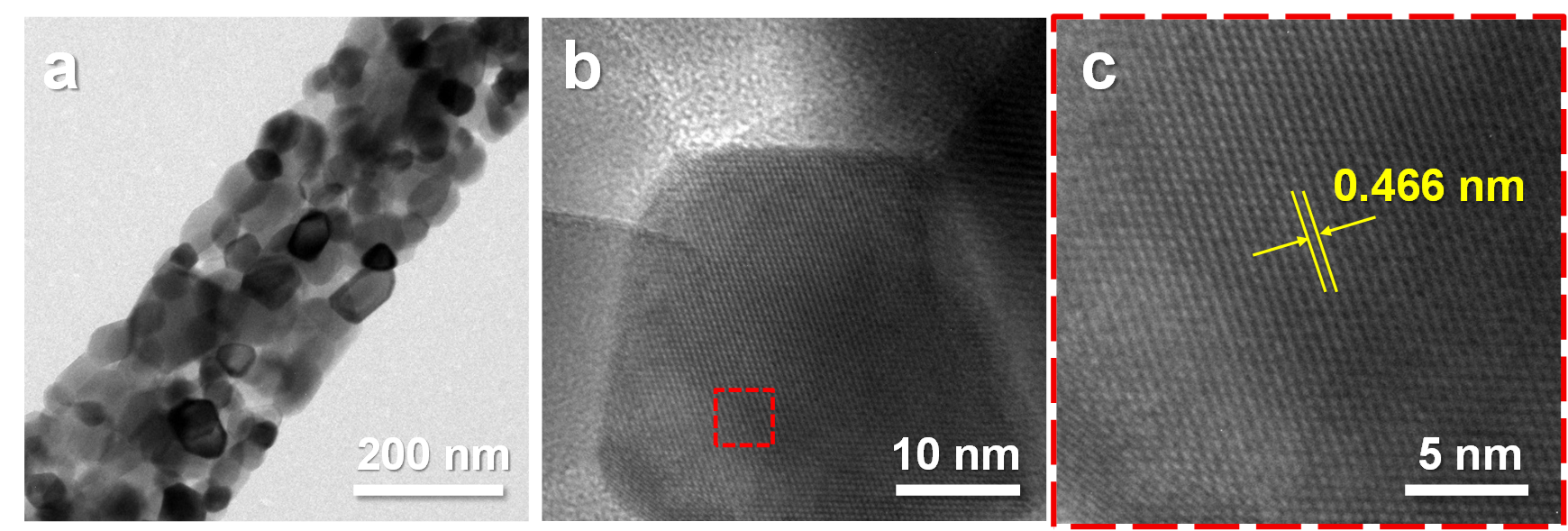
**

**Figure S1**. High resolution transmission electron microscopy (HRTEM) images of pristine Co_3_O_4_ NFs.

**[Figure S2]**

**Figure S2**. Scanning electron microscopy images of Co_3_O_4_ NFs@rGO with various concentration of graphene oxide (GO) (weight ratio of Co_3_O_4_ NFs and GO = (a) 30:1, (b) 16:1, (c) 12:1).

**[Figure S3]**

**
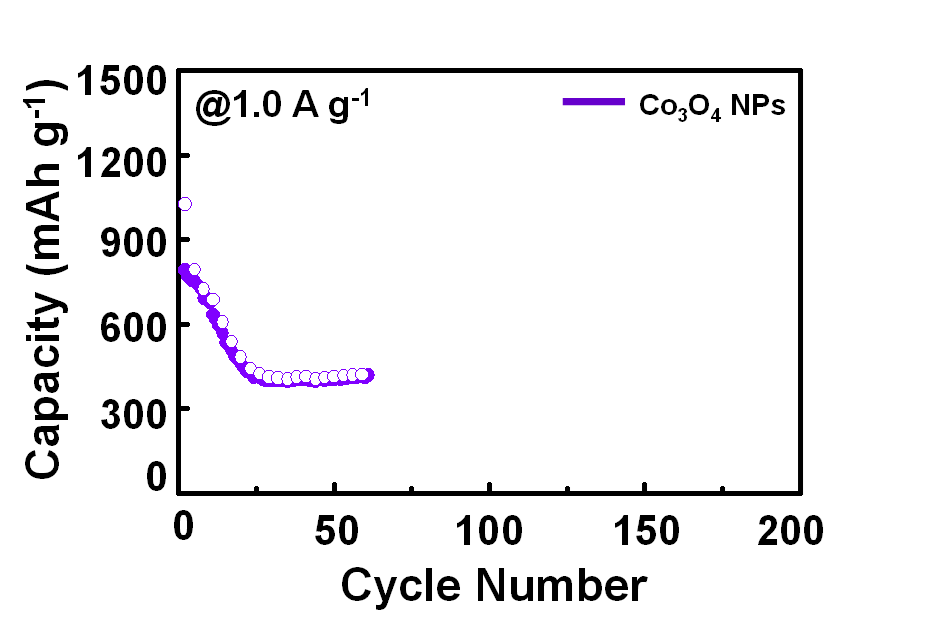
**

**Figure S3.** Electrochemical performance of pristine Co_3_O_4_ NP at current density of 1.0 A g^-1^.

**[Table S1]**

**Table S1.** Result of element analysis of Co_3_O_4_ NFs@rGO.

|  | **Nitrogen** | **Carbon** | **Hydrogen** |
| --- | --- | --- | --- |
| **1st** | **0.073** | **3.68** | **0.201** |
| **2nd** | **0.044** | **3.43** | **0.200** |
| **Average** | **0.059** | **3.56** | **0.200** |

**[Table S2]**

**Table S2.** Comparison of the electrochemical performance of Co_3_O_4_ anode for LIBs.

| **Structure**  **of Co_3_O_4_** | **Carbon** | **Carbon**  **contents**  **(wt%)** | **Capacity**  **(mAh g^-1^)** | **Current**  **density**  **(A g^-1^)** | **Cycle** | **Ref.** |
| --- | --- | --- | --- | --- | --- | --- |
| **Nanowires** | **Graphene** | **33.4** | **812** | **1.0** | **230** | **[1]** |
| **Hollow nanofibers** | **Carbon doping** | **6.0** | **1121** | **0.2** | **100** | **[2]** |
| **Porous cube** | **Graphene** | **27.2** | **980** | **0.2** | **80** | **[3]** |
| **Microrods** | **Graphene** | **44.4** | **550** | **0.089** | **70** | **[4]** |
| **Hierarchical tube** | **Carbon nanotube** | **13.0** | **577** | **4.0** | **200** | **[5]** |
| **Hollow spheres** | **Graphene** | **23.8** | **600** | **1.0** | **500** | **[6]** |
| **Nanowires** | **Carbon shell** | **18.0** | **989** | **0.445** | **50** | **[7]** |
| **Nanoparticles** | **Graphene** | **10.0** | **740** | **0.2** | **60** | **[8]** |
| **Nanoparticles** | **Carbon aerogel** | **75.0** | **478** | **0.05** | **50** | **[9]** |
| **Nanoparticles** | **Porous carbon** | **42.4** | **1060** | **0.1** | **100** | **[10]** |

**[Table S3]**

**Table S3.** Fitted electrochemical impedance component values of the Co_3_O_4_ NFs and the Co_3_O_4_ NFs@rGO.

|  | **R_E_ (Ω)** | **R_SEI_ (Ω)** | **R_CT-1_ (Ω)** | **R_CT-2_ (Ω)** | **R_P_ (Ω)** |
| --- | --- | --- | --- | --- | --- |
| **Co_3_O_4_ NFs** | 2.28 | 21.75 | 68.68 | 451.7 | 9.06 |
| **Co_3_O_4_ NFs@rGO** | 3.09 | 8.21 | 18.26 | 544.2 | 15.16 |

REFERENCES

1. Yao, X. *et al.* Synergistic Effect of Mesoporous Co_3_O_4_ Nanowires Confined by N-Doped Graphene Aerogel for Enhanced Lithium Storage. *Small* **12**, 3849–3860 (2016).
2. Yan, C., Chen, G., Zhou, X., Sun, J. & Lv, C. Template-Based Engineering of Carbon-Doped Co_3_O_4_ Hollow Nanofibers as Anode Materials for Lithium-Ion Batteries. *Adv. Funct. Mater.* **26**, 1428–1436 (2016).
3. Geng, H. *et al.* Porous cubes constructed by cobalt oxide nanocrystals with graphene sheet coatings for enhanced lithium storage properties. *Nanoscale* **8**, 7688–7694 (2016).
4. Tong, X., Zeng, M., Xu, H. & Li, J. Synthesis and lithium storage performance of graphene/Co_3_O_4_ microrods hybrids. *J. Mater. Sci. Mater. Electron.* **27**, 7657–7664 (2016).
5. Chen, Y. M., Yu, L. & Lou, X. W. D. Hierarchical Tubular Structures Composed of Co_3_O_4_ Hollow Nanoparticles and Carbon Nanotubes for Lithium Storage. *Angew. Chemie Int. Ed.* **55**, 5990–5993 (2016).
6. Sun, H., *et al.* Graphene-Wrapped Mesoporous Cobalt Oxide Hollow Spheres Anode for High-Rate and Long-Life Lithium Ion Batteries. *J. Phys. Chem. C* **118**, 2263–2272 (2014).
7. Chen, J. *et al.* Co_3_O_4_–C core–shell nanowire array as an advanced anode material for lithium ion batteries. *J. Mater. Chem.* **22**, 15056-15061 (2012).
8. Li, B. *et al.* Co_3_O_4_@graphene Composites as Anode Materials for High-Performance Lithium Ion Batteries. *Inorg. Chem.* **50**, 1628–1632 (2011).
9. Hao, F., Zhang, Z. & Yin, L. Co_3_O_4_/Carbon Aerogel Hybrids as Anode Materials for Lithium-Ion Batteries with Enhanced Electrochemical Properties. *ACS Appl. Mater. Interfaces* **5**, 8337-8344 (2013).
10. Wang, L. *et al.* Nitrogen-Doped Porous carbon/Co_3_O_4_ Nanocomposites as Anode Materials for Lithium-Ion Batteries. *ACS Appl. Mater. Interfaces* **6**, 7117–7125 (2014).
